# Safeguarding the Medical School

**Published:** 1917-03

**Authors:** 


### Safeguarding the Medical School.

The Texas Senate rejected the appointment of Dr. D. H. Lawrence, of El Paso, as a member of the Board of Regents of the University of Texas. There was no objection to the educational or moral qualifications of Dr. Lawrence, but it is understood that his rejection was due to an existing fear that in some way his connection with the board would result in trouble for the Medical Department of the University. The Senate received numerous appeals from medical men and others throughout the State, asking that the appointment of Dr. Lawrence should not be confirmed. All of these, it is said, resulted from the fear that harm might come to the University, and especially to the Medical Department should Dr. Lawrence have been given a place on the board.

The TEXAS MEDICAL JOURNAL does not know Dr. Lawrence personally, and has no opinion as to whether the fears as to his attitude to the Medical Department were well founded, but if there lurked in the appointment any semblance of danger to that valuable branch of the University the Senate did well in rejecting the appointment. Those who know have given their strongest approval to the medical school, and it today ranks among the very highest medical colleges in the United States—a rank that is due directly to the efficient management of those in charge and to the recognized ability of the members of the faculty. A fight on the Medical Department of the University of Texas, as it exists today, is an attack on medical education, not only in Texas, but in the United States. To do anything to lower the standard of medical education in Texas would reflect on the physicians of the entire State. Naturally, they arose almost in a body and appealed to the Senate of Texas not to permit any danger of this kind if it could be avoided, for Texas medical men are proud of their medical college and of its management.

It is hoped that the Senate will be as cautions in safeguarding other schools of the University. The men who planned the building of a great State here planned also for a "first-class University," and to allow that institution to be crippled in any way now would be to thwart the expressed wishes of the founders of the State, and to discredit the intelligence of its people. Texas has long enough been blighted by a bad name, by a reputation that it has not deserved, and the State cannot afford to have the work of its leading institution of learning ruined by political feeling of any kind. The JOURNAL has an abiding faith that this will not be permitted. Certainly too much has been wrought here in educational upbuilding for the State to take a backward step.

Dr. Lawrence may have been judged unjustly by the Senate of Texas, as well as by the members of the medical profession who opposed his confirmation, but under the existing fears and doubts nothing was left for the Senate to do but to say that it would by its course endeavor to obviate any possibility of danger to the Medical Department of the University, a course which should be pursued wherever and whenever there is a possibility that any part of the educational system of the State may suffer. The interests of the whole people are above those of any individual or group of individuals.

